# ecBSU1: A Genome-Scale Enzyme-Constrained Model of *Bacillus subtilis* Based on the ECMpy Workflow

**DOI:** 10.3390/microorganisms11010178

**Published:** 2023-01-11

**Authors:** Ke Wu, Zhitao Mao, Yufeng Mao, Jinhui Niu, Jingyi Cai, Qianqian Yuan, Lili Yun, Xiaoping Liao, Zhiwen Wang, Hongwu Ma

**Affiliations:** 1 Key Laboratory of Systems Bioengineering (Ministry of Education), Frontier Science Center for Synthetic Biology (Ministry of Education), Department of Biochemical Engineering, School of Chemical Engineering and Technology, Tianjin University, Tianjin 300072, China; 2 Biodesign Center, Tianjin Institute of Industrial Biotechnology, Chinese Academy of Sciences, Tianjin 300308, China; 3 National Technology Innovation Center of Synthetic Biology, Tianjin 300308, China; 4 Tianjin Medical Laboratory, BGI-Tianjin, BGI-Shenzhen, Tianjin 300308, China

**Keywords:** enzyme-constrained model, *Bacillus subtilis*, metabolic engineering

## Abstract

Genome-scale metabolic models (GEMs) play an important role in the phenotype prediction of microorganisms, and their accuracy can be further improved by integrating other types of biological data such as enzyme concentrations and kinetic coefficients. Enzyme-constrained models (ecModels) have been constructed for several species and were successfully applied to increase the production of commodity chemicals. However, there was still no genome-scale ecModel for the important model organism *Bacillus subtilis* prior to this study. Here, we integrated enzyme kinetic and proteomic data to construct the first genome-scale ecModel of *B. subtilis* (ecBSU1) using the ECMpy workflow. We first used ecBSU1 to simulate overflow metabolism and explore the trade-off between biomass yield and enzyme usage efficiency. Next, we simulated the growth rate on eight previously published substrates and found that the simulation results of ecBSU1 were in good agreement with the literature. Finally, we identified target genes that enhance the yield of commodity chemicals using ecBSU1, most of which were consistent with the experimental data, and some of which may be potential novel targets for metabolic engineering. This work demonstrates that the integration of enzymatic constraints is an effective method to improve the performance of GEMs. The ecModel can predict overflow metabolism more precisely and can be used for the identification of target genes to guide the rational design of microbial cell factories.

## 1. Introduction

*Bacillus subtilis* is a model organism of choice for the industrial production of various valuable compounds, such as biopolymers and proteins, due to its excellent capacity for protein secretion, good growth characteristics, distinct endogenous metabolism, and robustness in industrial fermentation [1]. Genome-scale metabolic network models (GEMs) of *B. subtilis* have been successfully applied to guide the production of riboflavin [2], isobutanol [2], 2,3-butanediol [3], and 3-hydroxypropionic acid [4]. The first *B. subtilis* GEM was published in 2008 [5], and several models were subsequently reported [2,6,7,8], which were updated in terms of reactions, metabolites and genes. The iBsu1147 model constructed by our team [2] has the highest number of reactions, metabolites and genes of all models reported to date (Figure S1). However, GEMs only consider stoichiometric constraints, making them unable to reflect the true state of the cell and locate kinetic bottlenecks limiting the flux through specific product synthesis pathways.

By contrast, enzyme-constrained models (ecModels) introduce enzyme kinetic information into a GEM, thus reflecting the protein resource limitation faced during cell growth, enabling them to identify the rate-limiting enzymes in the pathway and further guide rational metabolic engineering. As a consequence, ecModels have been successfully applied to guide the production of L-lysine [9], poly-glutamic acid [10], heme [11] and recombinant proteins [12]. Currently, three methods exist to automate the construction of ecModels, including GECKO [13], AutoPACMEN [14] and ECMpy [15]. GECKO is the earliest automated approach for the introduction of protein resource constraints into GEMs, which introduces average enzyme saturation coefficients and enzyme usage constraints from proteomic data [13]. However, GECKO adds many pseudo-metabolites representing enzymes, significantly increasing the complexity and scale of the model. Inspired by GECKO, Bekiaris et al. developed the AutoPACMEN automated workflow for the construction of ecModels, which introduces only one pseudo-reaction and pseudo-metabolite [14]. Recently, we developed the simplified Python-based workflow ECMpy, which allows the construction of an ecModel by directly adding a total enzyme amount constraint into a GEM [15]. Recently, ecModels have been constructed for several species, including *Escherichia coli* [9,12,15], *Saccharomyces cerevisiae* [13], *Aspergillus niger* [16], *Corynebacterium glutamicum* [17] and *B. subtilis* [10]. The first ecModel for *B. subtilis* (ec_iYO844) only integrated enzyme kinetic parameters for 17 reactions located in the central carbon metabolism using the GECKO method, but this model allowed more accurate prediction of the flux distribution and growth rate of wild-type and single-gene/operon deletion strains compared to the GEM [10].

In this study, we first systematically updated the iBsu1147 model through GPR update, biomass reaction standardization, etc., after which we established a comprehensive collection of parameters that affect the prediction accuracy of the ecModel (e.g., the enzyme kinetics data and quantitative information on enzyme subunit composition), and constructed the first genome-scale ecModel of *B. subtilis* (ecBSU1), using an updated ECMpy workflow [17]. Subsequently, we used ecBSU1 to accurately predict the growth rate of *B. subtilis* on different carbon sources, simulate the overflow metabolism, and explain the trade-off between biomass yield and enzyme usage efficiency. Finally, we predicted the target genes of *B. subtilis* for enhancing the production of industrial chemicals (e.g., riboflavin, menaquinone 7, and acetoin, etc.), and the predictions were in good agreement with the literature.

## 2. Methods

### 2.1. Model Update

The model iBsu1147, which has the most reactions and genes, was selected as the initial model for the integration of enzymatic constraints. Since the iBsu1147 model was released in 2013, we performed quality control on the model, covering substrate utilization, redox balance, energy balance, biomass reaction standardization, and mass balance. Our previous results shown that the *k*_cat_ and molecular weight (MW) of an enzyme affect the prediction accuracy of the ecModel [15]. For example, the correctness of the EC number affects the correctness of the corresponding *k*_cat_, and the GPR relationship affects the accuracy of MW calculation. Accordingly, we systematically corrected the EC number and GPR relationships. We used the GPRuler tool [18] and protein homology similarity to uncover the potential GPR errors present in the reaction (see [17] for details). In order to meet the requirements of AutoPACMEN processes for metabolic network format input, we converted most of the KEGG IDs and ModelSEED [19] IDs (both metabolites and reactions) into BiGG [20] IDs. Finally, we named the modified model iBsu1147^R^ (Revised iBsu1147).

### 2.2. Data Acquisition

The molecular weight (MW) of each enzyme was downloaded from the UniProt database according to the gene ID, and the quantitative subunit information was obtained by parsing the ‘Interaction information’ in UniProt [17]. For example, P39119 is described in UniProt as a ‘homodimer’, so its subunit number is 2 (all corresponding information is listed in Table S1). The *k_cat_* values were obtained from the BRENDA [21] and SABIO-RK [22] databases according to the EC numbers using AutoPACMEN. *B. subtilis* protein abundance data were obtained from the PAXdb [23] database, and enzyme mass fraction was calculated according to Equation (1):

(1) $$f=\sum_{i=1}^{p\_num}A_{i}MW_{i}/\sum_{j=1}^{g\_num}A_{j}MW_{j}$$

where $A_{i}$ and $A_{j}$ represented the abundance of the *i*-th protein (*p_num* represents proteins expressed in the model) and *j*-th protein (*g_num* represents proteins expressed in the whole proteome).

### 2.3. Construction of ecBSU1

**Figure 1:**
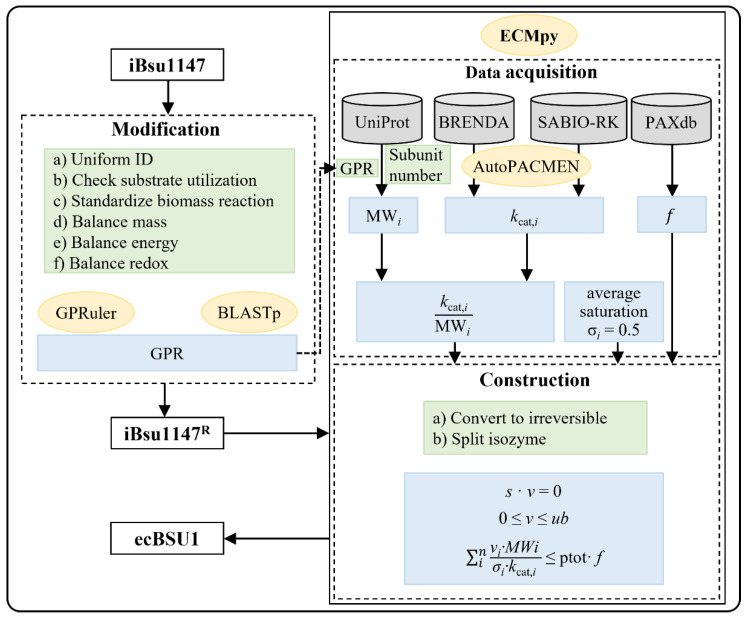
Workflow for the construction of ecBSU1.

The enzyme-constrained model was constructed based on iBsu1147^R^ following the ECMpy workflow and named ecBSU1 (Figure 1). Firstly, we divided reversible reactions in iBsu1147^R^ into pairs of irreversible reactions, and split reactions catalyzed by multiple isoenzymes into different reactions (append num in reaction ID, e.g., GLCpts_num1), so that each reaction only has one corresponding enzyme. Next, we calculated the MW of each enzyme. For reactions catalyzed by enzyme complexes, we used the total sum of proteins in the complex ($MW=\sum_{j=1}^{m}N_{j}*MW_{j}$, where m is the number of different subunits in the enzyme complex and N_j_ is the number of jth subunits in the complex). Finally, a new enzymatic constraint ($\sum_{i=1}^{n}\frac{v_{i}*MW_{i}}{\sigma_{i}*k_{cat,i}}\leq ptot*f$) was introduced into the model, where *ptot*, *f*, and $\sigma_{i}$ represent the total protein fraction in *B. subtilis*, the mass fraction of enzymes, and the saturation coefficient of the i-th enzyme, respectively (see [15] for details).

### 2.4. Calibration of Enzyme Kinetic Parameters

To improve the agreement of model predictions with experimental data, the enzyme-constrained model required further adjustment of the original *k_cat_* values, analogous to GECKO, AutoPACMEN and ECMpy. For model calibration, GECKO used a manual calibration approach (GECKO 2.0 updated to automatic calibration) and AutoPACMEN was a direct 10-fold expansion without an automated process. In contrast, ECMpy identify the most likely wrong parameters and corrected them through an automatic process based on the cost of enzymes. The process includes the following steps: First, we calculated the enzyme cost of each reaction in the pathway with biomass maximization as the objective (see [15] for details). Subsequently, we ranked the reactions by enzyme cost and selected the reactions with the largest enzyme costs as potential reactions that need correction. Next, we modified the reaction *k*_cat_ to the maximal corresponding *k*_cat_ in the BRENDA and SABIO-RK databases. We reiterated the above correction until the growth rate reached a reasonable steady state (For example, the experimentally reported values), as described in GECKO 2.0 [24].

### 2.5. Phenotype Phase Plane (PhPP) Analysis

Different rates of substrate uptake and oxygen supply affect the cellular metabolic phenotype, leading to different maximal growth rates. We performed PhPP analysis on iBsu1147^R^ and ecBSU1 as described in the literature [16] to obtain a global view of how optimal growth rates are affected by varying glucose and oxygen uptake rates. To implement the PhPP analysis, the reaction fluxes of oxygen and glucose were, respectively, varied in the range of 0–50 mmol/gDW/h and 0–15 mmol/gDW/h, after which pFBA calculations were performed with biomass maximization as the objective.

### 2.6. Prediction of Growth Rates on Different Carbon Sources

To evaluate the ability of ecBSU1 to predict phenotypes, we simulated the growth rate of *B. subtilis* on 8 different carbon sources, and compared the prediction results of iBsu1147^R^ and ecBSU1 with reported values [25]. Next, the model and experimental results were used to calculate the estimation error of the growth rate and normalized flux error (see [15] for detail).

### 2.7. Simulation of Overflow Metabolism

Overflow metabolism refers to the seemingly wasteful strategy in which cells use fermentation instead of the more efficient respiration to generate energy, despite the availability of oxygen [26]. As a result of employing this metabolic strategy, cells excrete (or “overflow”) metabolites such as lactate, acetate, and ethanol. We explored the overflow metabolism of the *B. subtilis* using ecBSU1 by setting the substrate uptake rate on a gradient of 0 to 10 mmol/gDW/h and solving for pFBA to maximize the biomass. To further explain the metabolic overflow phenomenon, we analyzed the biomass yield, enzyme usage efficiency, reaction enzyme cost, energy synthesis enzyme cost, and oxidative phosphorylation ratio (proportion of glucose used for the oxidative phosphorylation pathway to total glucose) (see [15,27] for details).

### 2.8. Prediction of Metabolic Engineering Targets

Compared to GEMs, ecModels can calculate enzyme costs in addition to reaction fluxes, which is useful for identifying key enzymes in the pathway [9]. In this work, we analyzed the enzyme cost of each reaction to identify kinetic bottleneck reactions (the reactions with the largest enzyme cost) (Equation (2)) by setting glucose as the substrate, the product as the objective, and the low bound of biomass reaction as 10% of the maximal growth rate, as described in the literature [9]. Finally, we selected the Top 15 reactions with the highest enzyme cost as potential metabolic engineering targets.

(2) $$Enzyme\;cost_{i}=\frac{v_{i}*MW_{i}}{\sigma_{i}*k_{cat,i}}$$

### 2.9. Software

The described ecBSU1 model generator is written in Python 3.6.5. Aside of Python’s standard library, the model generator also uses the modules biopython, cobrapy (version = 0.13.3) [28], scipy, openpyxl, and requests. The metabolic pathway simulations were performed using pFBA (parsimonious Flux Balance Analysis), which seeks to minimize the flux associated with each reaction in the model while maintaining optimum flux through the objective function [29].

## 3. Results

### 3.1. GPR Correction of iBsu1147

EC numbers affect the extraction and assignment of *k*_cat_ data in the workflow, ultimately affecting the prediction accuracy of the ecModel. Consequently, we replaced the old EC numbers in the model based on BRENDA, updating a total of 38 reactions (Table S2). MW is also an important factor affecting the prediction accuracy of ecModels. Two major factors influence the final MW of the enzyme assigned to a specific reaction: whether the protein is composed of subunits (GPR relationship) and the number of each subunit. We systematically corrected the GPR relationships in the model by referring to the methods used in ecCGL1 (GPRuler tool and protein homology similarity) [17]. We first identified 146 reactions containing protein complex information using the GPRuler tool, 80 of which were consistent with the model. For the remaining 66 reactions in the model that contained “and” relationships, we performed a manual check using the UniProt, KEGG, and BioCyc databases, and found that 27 reactions were correct in the model, 6 reactions were correct in the GPRuler tool, and the remaining 33 reactions were incorrect in both (Table S3). For example, the GPR relationship of the ribose ABC transport system is ‘*BSU35930* and *BSU35940* and *BSU35950* and *BSU35960′* in the model, but the GPRuler tool did not include ‘*BSU35930′*. By searching UniProt, we found that ‘*BSU35930′* encodes D-ribose pyranase, which catalyzes the interconversion of beta-pyran and beta-furan forms of D-ribose, indicating that the result of GPRuler tool is correct. In addition, when verifying the results of the GPRuler tool, some reactions in the model were modified based on the annotation information of the proteins in the databases, including the deletion of 7 reactions (e.g., PDHbr and AKGDb), the addition of 4 new reactions (e.g., AKGDH and FCLT3), and the modification of GPR relationships for 5 reactions (e.g., RBFSb and 26DPAi).

We also observed that there were 58 reactions in the model for which the “and” relationships were not identified by the GPRuler tool, and only 9 reactions had more than 25% similarity (Table S4). By searching the database, we found that 7 reactions needed to be corrected, 3 of which needed to be changed from “or” to “and” relationships, and 4 in which the proteins needed to be replaced (Table S4). For example, the GPR relationship for NADH-dependent butanol dehydrogenase (BTS) is ‘*BSU31360* and *BSU31370′*, which has 74% sequence similarity. By further searching the BioCyc database for verification, we found that the protein encoded by *BSU31370* is a bifunctional enzyme that catalyzes two reactions (NADPH-dependent furan aldehyde reductase and NADPH-dependent butanol dehydrogenase), whereas the protein encoded by *BSU31360* catalyzes only the NADPH-dependent butanol dehydrogenase reaction. They are more likely to be two isoenzymes, and therefore the correct GPR relationship for BTS should be ‘*BSU31360* or *BSU31370′*. Finally, 1736 reactions, 1459 metabolites, and 1155 genes were included in iBsu1147^R^.

### 3.2. Other Modifications of iBsu1147

After running quality control of the iBsu1147^R^ model in terms of substrate utilization, redox balance, energy balance, biomass reaction standardization, and mass balance, we found that all these aspects led to abnormalities in the metabolic pathways generated by the simulation. The boundary of 6 reactions was modified in terms of substrate utilization (e.g., EX_chor_e and MALt2r). For example, experiments have shown that *B. subtilis* can grow using malate [25], so the upper and lower boundaries of the malate transport reaction (MALt2r) in the model should not be 0. From a reducing power perspective, we modified the catalytic orientation of 4 reactions (e.g., NODOx and P5CR) based on BioCyc [30] to avoid pathway calculation errors. For example, P5CR (1pyr5c_c + h_c + nadph_c –> nadp_c + pro__L_c) is reversible in the model, but a search by BioCyc revealed that the reaction acts as the final step of the L-proline synthesis pathway I, which is unidirectional. In addition, the catalytic orientations of 3 respiratory chain-related reactions were also modified (e.g., CYOR3m and CYTB_B2). In total, 13 reactions in the model were corrected for the boundaries (Table S5).

In addition, the molar mass of biomass and its components (e.g., proteins, nucleic acids, etc.) was 1 g/mmol, and deviations from this value will result in errors in the calculated specific growth rate. Using the BiomassMW algorithm [31], we examined the biomass equation of the iBsu1147^R^ model and found that the original molecular mass of the biomass was 1.025 g/mmol, and H^+^ was missing in the right side of the biomass equation (the coefficient was 105) produced by the hydrolysis of ATP for growth-associated maintenance energy. After correction, the molar mass of the biomass became 0.92 g/mmol. Subsequently, we examined the precursor metabolites and found that the molar masses of protein, DNA, and RNA were 0.86 g/mmol, 0.95 g/mmol, and 0.95 g/mmol, respectively. We normalized the coefficients so that the molar masses of all components were 1 g/mmol, and the molar masses of the biomass also became 1 g/mmol. The details of all the modified reactions are listed in Table S6.

Finally, we found that iBsu1147^R^ contains different IDs for metabolites and reactions, including KEGG IDs and ModelSEED IDs. To meet the input requirements of AutoPACMEN, we converted the KEGG IDs of 1007 metabolites and 785 reactions, as well as the ModelSEED IDs of 265 metabolites and 542 reactions into BiGG IDs. In addition, we kept the original IDs for reactions and metabolites that were not included in the BiGG database.

### 3.3. Basic Information of ecBSU1

**Figure 2:**
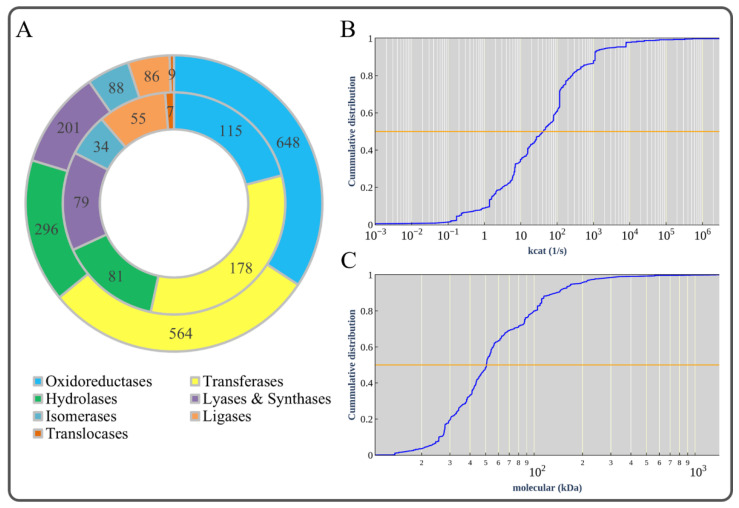
Basic information of ecBSU1. (**A**) Enzyme classification. The outer ring indicates that there were 1892 reactions with enzyme kinetic data, which can be divided into seven categories. The inner ring indicates that these reactions included 549 kinds of enzymes according to the corresponding EC numbers, which can also be divided into seven categories. (**B**) Cumulative distribution of *k*_cat_ values. (**C**) Cumulative distribution of molecular weights.

We used AutoPACMEN to match 2331 *k_cat_* values (439 were obtained by filling, see Table S7) for 3307 reactions (splitting of reversible reactions and isozymes), accounting for 70.5% of the total reactions and 76.4% excluding exchange reactions. In total, 1892 reactions were catalyzed by enzymes with 549 different EC numbers, among which oxidoreductases and transferases accounted for the majority (Figure 2A, inner ring). These *k_cat_* values spanned 9 orders of magnitude, with a median value of 46.17 s^−1^ (Figure 2B). In total, the molecular weights for 1155 enzymes were obtained from UniProt based on the corresponding gene IDs, covering 3 orders of magnitude, with a median value of 50.41 kDa (Figure 2C). The enzyme mass fraction *f* was calculated from the proteomic data in the PAXdb. For *B. subtilis*, we chose the dataset “*B. subtilis*-Whole organism (Integrated)” with the highest measurement coverage and evaluation score, and calculated *f* = 0.588 g enzyme/g protein according to Eq. 1. Finally, the initial *B. subtilis* ecModel (ecBSU1) contained 1155 genes, 1459 metabolites, 3307 reactions, and 2331 enzyme kinetic parameters, with a total enzyme bound of 0.165 g enzymes/gDW.

### 3.4. Correction of Enzyme Kinetic Parameters to Overcome Model Over-Constraint

**Figure 3:**
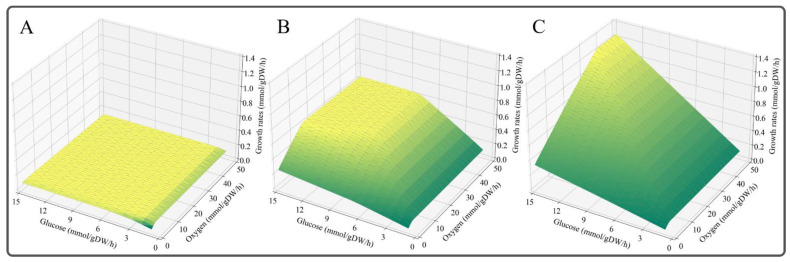
The solution space of iBsu1147^R^ and ecBSU1. Changes in the maximal growth rate with the increase of glucose and oxygen uptake rates in ecBSU1 before *k_cat_* correction (**A**), ecBSU1 after *k_cat_* correction (**B**), and iBsu1147^R^ (**C**).

Over-constraint in the initial ecModel is normal due to some reactions with abnormal *k*_cat_ values (usually too low), as was reported for ecYeast7 [13], eciML1515 [9], eciJB1325 [16] and eciJO1366 [32], which all needed *k_cat_* correction. We found that the maximal growth rate calculated by ecBSU1 with glucose as substrate was 0.092 h^−1^ (Figure 3A), which was significantly lower than the experimental value of 0.59 h^−1^ [25]. To overcome this over-constraint, we modified the *k_cat_* values of the reactions with the largest enzyme cost to the corresponding maximal *k_cat_* values in BRENDA and SABIO-RK. After modifying 28 reactions (Table S8), the maximal growth rate on glucose reached 0.612 h^−1^ (Figure 3B), which was consistent with the experimental observations. However, the growth rate predicted by iBsu1147^R^ increased linearly with increasing carbon source consumption (Figure 3C), which is unrealistic. In addition, PhPP analysis showed that the solution space of ecBSU1 was significantly reduced compared with the metabolic network model (Figure 3B,C). These results demonstrated that incorporating enzymological constraints into a GEM can reduce the flux solution space and enable the model to simulate a more realistic cellular phenotype.

### 3.5. Simulation of Overflow Metabolism

**Figure 4:**
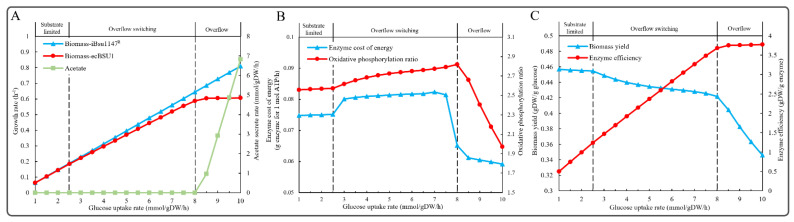
Simulation of overflow metabolism. (**A**) Comparison of in silico overflow between iBsu1147^R^ and ecBSU1. (**B**) Enzyme cost of energy metabolism and oxidative phosphorylation ratio. (**C**) Trade-off phenomenon simulated by ecBSU1.

Enzyme-constrained models have been used to simulate overflow metabolism in *S. cerevisiae* [13,33], *C. glutamicum* [17], and *E. coli* [15]. It has been reported in the literature that *B. subtilis* exhibits overflow metabolism in the presence of excess glucose [34]. We explored the phenomenon of overflow metabolism in *B. subtilis* using ecBSU1, and the simulation results showed that at a high glucose uptake rate of 8 mmol/gDW/h, *B. subtilis* was indeed able to engage metabolic overflow into acetate at high glucose uptake rate (Figure 4A). By contrast, in iBsu1147^R^, glucose increased linearly with the growth rate and could not simulate the phenomenon of overflow metabolism (Figure 4A). We then calculated the energy synthesis enzyme cost and oxidative phosphorylation ratio to explore the pathway adjustment strategy of overflow metabolism in *B. subtilis*. The simulation results indicated that at high growth rates, the acetate-producing fermentation pathway was activated to maintain growth due to its low enzyme cost in comparison with the energetically efficient respiratory oxidative phosphorylation pathway (Table S9 and Figure 4B).

In order to further explain the metabolic overflow phenomenon, we analyzed the biomass yield and enzyme usage efficiency at different glucose uptake rates. As shown in Figure 4C, there was a clear trade-off between yield and enzyme usage efficiency, so that the metabolic processes could be divided into a substrate-limited stage, overflow switching stage, and overflow stage. At the substrate-limited stage, the glucose uptake rate was low (less than 2.5 mmol/gDW/h) and did not reach the constraint of protein resources, resulting in a substrate uptake rate proportional to the growth rate. At the overflow switching phase (between 2.5 and 8 mmol/gDW/h), the cells redistributed the metabolic fluxes toward pathways with high enzyme usage efficiency but low biomass yield. Finally, overflow metabolism occurred in the overflow stage (greater than 8 mmol/gDW/h). That means at a high glucose uptake rate (8 mmol/gDW/h), *B. subtilis* needs to activate a fermentation pathway with low energy production efficiency but high enzyme efficiency to maintain growth, resulting in the overflow to acetate (Figure 4A–C).

### 3.6. Enzyme-Constrained Integration Improved the Phenotype Prediction

**Figure 5:**
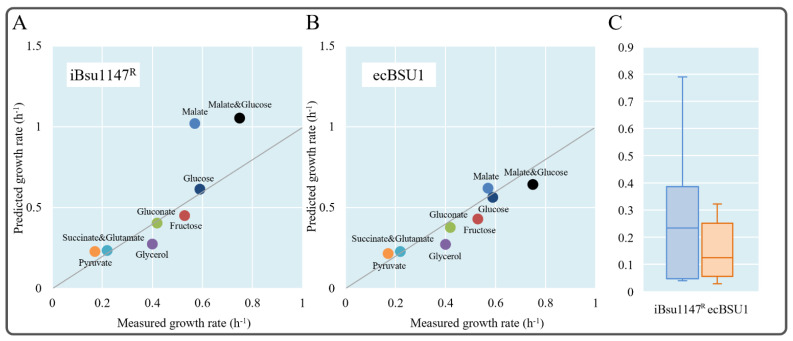
Predicted *B. subtilis* growth rates on different carbon sources using iBsu1147^R^ (**A**) and ecBSU1 (**B**). (**C**) Distribution of prediction errors of internal fluxes from iBsu1147^R^ and ecBSU1.

To further test the enzyme-constrained model, we simulated the growth rates of *B. subtilis* on 8 different carbon sources reported in the literature [25], and compared the prediction results of iBsu1147^R^ and ecBSU1 (Table S10). As shown in Figure 5A,B, the predicted growth rates of ecBSU1 were lower than those of iBsu1147^R^ due to the introduction of enzymatic constraints. Especially for the two experiments using malate alone or malate and glucose as substrates, the prediction results of iBsu1147^R^ were unreasonably higher than the experimental results (Figure 5A). By contrast, the predicted rates of ecBSU1 were closer to the experimental values (Figure 5B,C). For all other carbon sources, the prediction results of ecBSU1 were also similar to or better than those from iBsu1147^R^. Moreover, we identified errors in some reactions of ec_iYO844 (the first ecModel for *B. subtilis*), as 17 reactions with *k_cat_* values were unidirectional, resulting in its ability to simulate growth using only glucose as a substrate (Figure S2).

Further in-depth analysis using the ecModel revealed that the addition of the enzyme kinetic constraint information allowed ecModel to simulate the overflow of by-products from cells at high substrate concentrations. For example, the ecBSU1 results showed that it severe overflow metabolism would occur when utilizing malate at 26.51 mmol gDW^−1^ h^−1^, producing 16.39 mmol gDW^−1^ h^−1^ of acetate, thus predicting a biomass growth rate (0.618 h^−1^) close to the experimental value [35]. However, although the growth rate was predicted accurately, the overflow products were different from the experimental results (9.50 mmol gDW^−1^ h^−1^ acetate and 3.93 mmol gDW^−1^ h^−1^ pyruvate). The difference in overflow products is mainly due to the limitations of the current ecModel, as the optimization process of the model only supports the pathway that generates the lowest enzyme cost, so that the current ecModel will only generate one overflow product.

### 3.7. The Enzyme-Constrained Model Predicted Target Genes for Improving the Production of Chemicals

**Figure 6:**
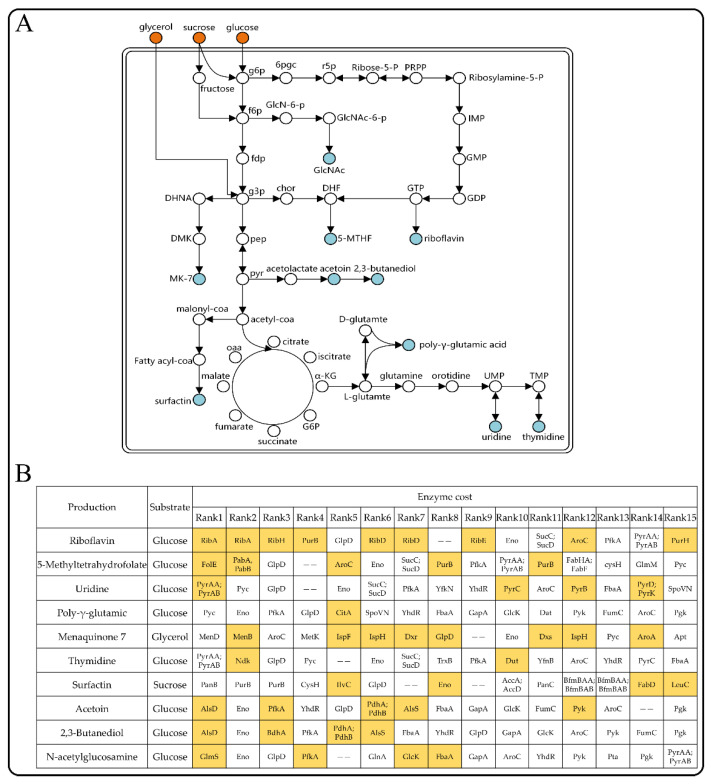
Predicted metabolic engineering targets for improving the synthesis of the indicated products in *B. subtilis*. (**A**) The synthesis pathways of 10 products in *B. subtilis*. (**B**) Prediction of targets for the synthesis of the 10 products in *B. subtilis* using ecBSU1. Targets that have been reported in the literature are marked in yellow, and those that require multiple genes to act together are marked with ‘‘--’’.

GEMs play a guiding role in predicting metabolic engineering targets. In this work, we predicted potential target genes for the synthesis of several important chemicals (e.g., riboflavin, menaquinone 7, acetoin, etc.) in *B. subtilis* based on the enzyme cost of reactions. We selected 10 products for analysis from 51 commercial chemicals produced using the *B. subtilis* platform summarized in the literature [36]. The products were classified according to the need to introduce exogenous reactions, and the location of the precursor in the central metabolic pathway (Figure 6A). We set glucose as the substrate and fixed the growth rate at 0.06 h^−1^ (10% of the maximum) and performed reaction enzyme cost calculations with each of these 10 products as the objective function, respectively. Subsequently, a literature search was performed to validate the top 15 reactions in terms of enzyme cost in each pathway.

We found that most of the predicted potential targets for the 10 products have been reported in the literature (Figure 6B, Table S11). Among them, riboflavin and menaquinone 7 covered the most targets, with more than half of the predicted 15 potential targets (9 and 8, respectively) having been reported in the literature (Figure 6B). Notably, enzymes with the highest enzyme cost in the synthetic pathways of riboflavin and uridine are GTP cyclohydrolase II (encoded by *ribA*, ru5p__D_c --> db4p_c + for_c + h_c) and carbamoylphosphate synthetase (encoded by *pyrAA* and *pyrAB*, 2.0 atp_c + gln__L_c + h2o_c + hco3_c --> 2.0 adp_c + cbp_c + glu__L_c + h_c + pi_c), respectively (Table S10). Experiments have been performed to show that both enzymes are rate-limiting enzymes for their respective products [37,38,39]. For example, studies on a riboflavin production strain of *B. subtilis* showed that the insertion of an additional copy of *ribA* led to improved riboflavin titers and yields on glucose of up to 25% [37]. In addition, Wang et al. released the feedback inhibition of carbamoylphosphate synthetase encoded by *pyrAB*, leading to a 245% increase of uridine production, whereby the conversion of glucose to uridine increased by 10.5%, while overexpression of the *pyr* operon increased the production of uridine by a further 31% [38]. For 5-methyltetrahydrofolate, GTP cyclohydrolase 1 (encoded by *folE,* gtp_c + h2o_c --> ahdt_c + for_c + h_c) caralyzes a limiting step for the synthesis of the important precursor dihydrofolate (DHF), and co-overexpression of *folC*, *pabB*, *folE*, and *yciA* resulted in an additional 66.8-fold improvement of the 5-methyltetrahydrofolate titer, which reached 960.27 μg/L [40]. Therefore, we can speculate that the reactions with the highest enzyme cost in the pathways of several other products (e.g., PanB for surfactin, Pyc for poly-γ-glutamic acid, MenD for menaquinone 7, etc.) are likely to be bottleneck reactions and potential targets for metabolic engineering.

## 4. Discussion

During the construction of traditional GEMs, not much attention is paid to whether the GPR relationship is “and” or “or”, but the correctness of these two relationships directly affects the simulation accuracy of ecModels. In this study, we systematically examined and corrected the GPR relationships in iBSU1147 by combining the GPRuler tool and protein homology. In addition, we systematically updated the EC numbers, carbon source utilization pathways, biomass reactions, mass balance, energy balance, and redox balance in the iBsu1147 model. This quality checking and correction process has far-reaching implications for improving the quality of GEMs and the construction of high-quality ecModels.

Using iBsu1147^R^, we constructed ecBSU1 based on the ECMpy approach, in which 2331 of 3307 reactions were integrated with enzyme constraints according to AutoPACMEN, and the *k*_cat_ coverage reached 76.4% after excluding 254 exchange reactions. The coverage of enzyme parameters was much higher than in ec_iYO844, which integrated only 17 reactions (1.67% of the total number of reactions) located in the central carbon metabolism with the addition of *k_cat_* values. However, enzyme kinetic data are sparse, and measured turnover rates are normally available for only a small fraction of metabolic reactions even in well-studied organisms [41]. Therefore, even though we covered the *k_cat_* data for most of the reactions in the model, only 163 reactions had *k_cat_* values derived from *B. subtilis*.

Due to the diversity and incomplete coverage of enzyme parameters in the database, the initial ecModel was over-constrained, but it was able to accurately predict cellular phenotypes after *k*_cat_ correction for 28 reactions. The ecBSU1 corrects the problem that the growth rate of GEMs increases indefinitely with the increase of the carbon source utilization rate. Next, ecBSU1 and iBsu1147^R^ were, respectively, used to simulate the growth rate of *B. subtilis* on different substrates, and the results showed that the accuracy of ecBSU1 was much better than that of iBsu1147^R^ at the high growth rate stage with enzyme constraints. In addition, the overflow metabolism of *B. subtilis* was explored using ecBSU1, which showed a physiological state of overflow metabolism in the presence of excess substrates, and demonstrated a trade-off between biomass yield and enzyme usage efficiency.

Thus, ecBSU1 can be used to guide the rational design of microbial cell factories from a new perspective. The simulation results of GEMs usually only contain reaction fluxes, which cannot distinguish the physiological characteristics of each reaction. By contrast, ecBSU1 combines the kinetic characteristics of each reaction based on fluxes, thus demonstrating the enzyme consumption of each reaction, which can assist us to locate the kinetic bottlenecks of different metabolic states. We simulated the enzyme consumption of *B. subtilis* for the synthesis of several commercial chemicals, and the reactions with the highest enzyme consumption were identified as metabolic engineering targets, which was in good agreement with the literature. This provides a new strategy and theoretical basis for metabolic engineering.

Currently, ecBSU1 has a typical limitation also found in other ecModels, as the implementation of the enzyme abundance constraint is highly dependent on precise kinetic parameters and abundance data for each enzyme [14], both of which are often inadequate. Although AutoPACMEN and GECKO adopt automated strategies to supplement the missing data by fuzzy matching to similar reactions or organisms (based on the EC number and substrate), this can cause model predictions to deviate significantly from experimental observations [42]. Machine learning or deep learning tools are valuable for uncovering global trends of enzyme kinetics and physiological diversity, which can further elucidate the details of a large-scale ecModel [43,44]. For example, Li et al. provided a deep learning approach (DLKcat) for high throughput *k*_cat_ prediction for metabolic enzymes from any organism merely from substrate structures and protein sequences [44]. Using this approach, they predict genome-scale *k*_cat_ values for more than 300 yeast species. In addition, the integration of enzyme constraints greatly improves the predictive power of GEMs and brings the model predictions closer to the experimental measurements, but the biological system is too complex and to be fully described by enzymatic constraints alone. Therefore, it is necessary to integrate more biological data into novel composite constraints, which can include data on thermodynamics [45,46] and regulatory networks [47], or construct a whole-cell GEM [48].

## 5. Conclusions

This work integrated enzymatic constraints into the GEM of *B. subtilis* on a genome-wide scale, which significantly improved its metabolic phenotype prediction ability. The resulting model can be used to explain metabolic overflow phenomena and predict metabolic engineering targets for the synthesis of commercial chemicals in *B. subtilis*. This study has guiding significance for the rational design of microbial cell factories and provides an important integrated metabolic network model of *B. subtilis*. Finally, it also offers insights for the improvement of GEMs of other strains, so that the role of such models in the development of synthetic biology can be broadened in the future.

## Data Availability

The scripts and datasets generated during and/or analyzed during the current study can be found at: https://github.com/tibbdc/ecBSU1 (accessed on 7 January 2023).

## Supplementary Materials

The following supporting information can be downloaded at: https://www.mdpi.com/article/10.3390/microorganisms11010178/s1, Figure S1: Model development of *B. subtilis*; Figure S2: Growth rates of *B. subtilis* on different carbon sources predicted using ec_iYO844; Table S1: Correspondence between subunit descriptions and subunit numbers in UniProt; Table S2: EC number modification; Table S3: GPR modifications based on the GPRuler tool; Table S4: GPR modification based on homology; Table S5: Modification of reaction boundaries; Table S6: Biomass reaction standardization and mass balance using BiomassMW; Table S7: *k*_cat_ in ecBSU1; Table S8: Reactions for *k*_cat_ calibration; Table S9: Enzyme cost of energy metabolism; Table S10: Growth rates on different carbon sources predicted using iBsu1147^R^ and ecBSU1; Table S11: Target prediction based on enzyme cost.
