# Supplementary figures and images for: ecBSU1: A Genome-Scale Enzyme-Constrained Model of Bacillus subtilis Based on the ECMpy Workflow
Source: Microorganisms. 2023 Jan 11;11(1):178. doi: 10.3390/microorganisms11010178 (PMC9864840; doi:10.3390/microorganisms11010178)

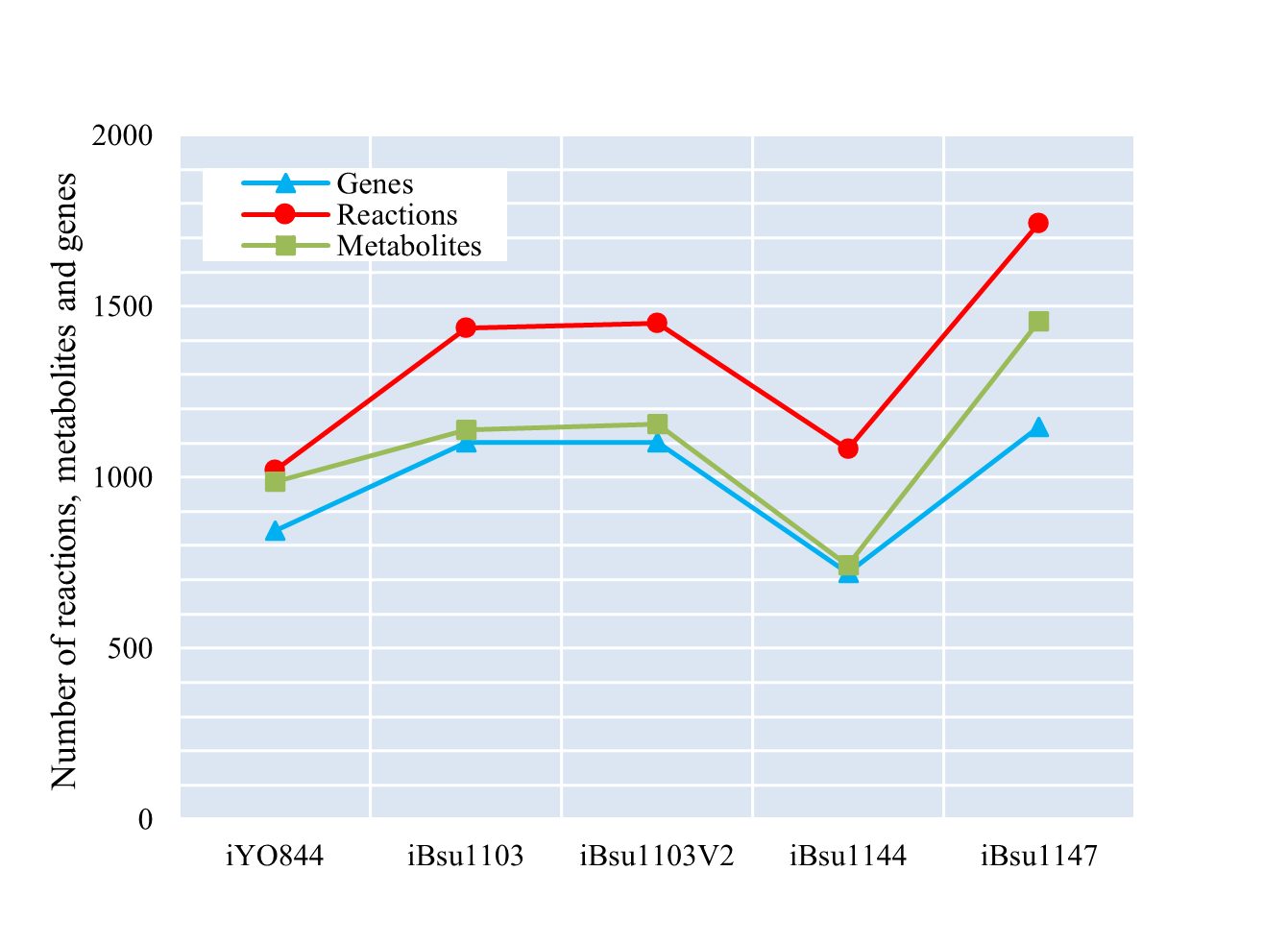

Supplement: Supplementary file 1 [file microorganisms-11-00178-s001.zip › Figure S1.tif]

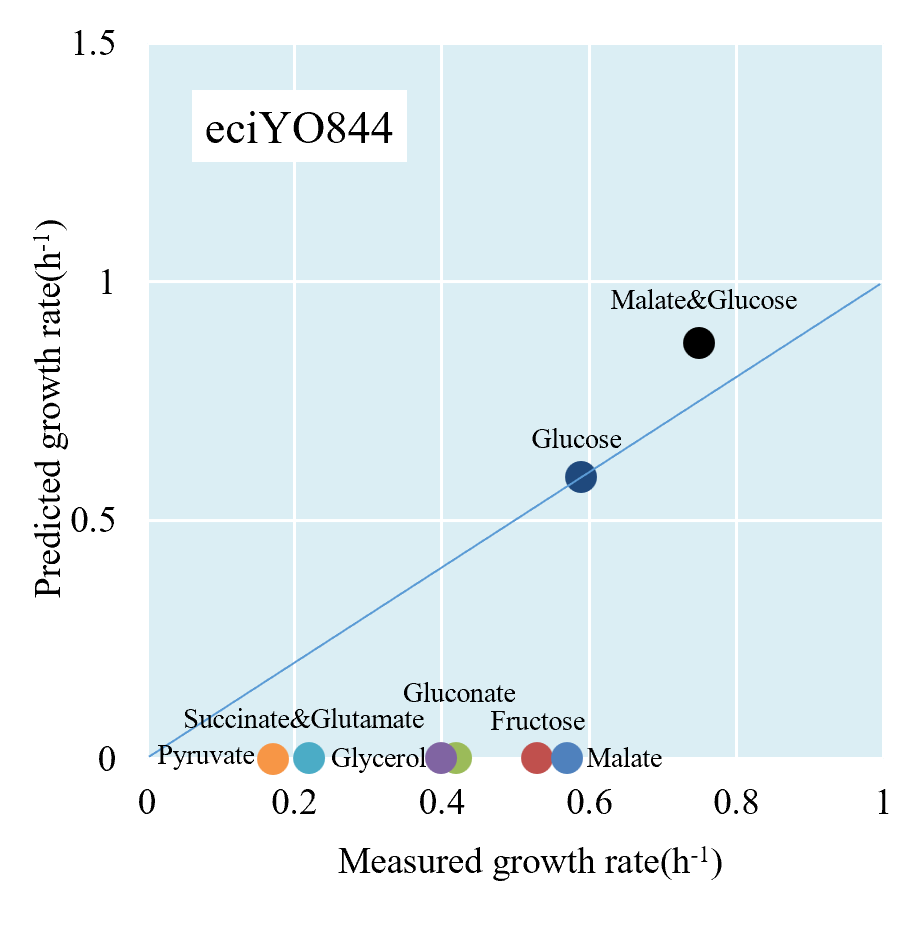

Supplement: Supplementary file 1 [file microorganisms-11-00178-s001.zip › Figure S2.tif]
